# Supplementary material for: The interactome of intact mitochondria by cross-linking mass spectrometry provides evidence for coexisting respiratory supercomplexes
Source: Mol Cell Proteomics. 2017 Dec 8;17(2):216–32. doi: 10.1074/mcp.RA117.000470 (PMC5795388; doi:10.1074/mcp.RA117.000470)
Supplement: Supplemental Data [file supp_RA117.000470_133922_0_supp_23978_fzffwf.zip › spectra_annotation/mito_DR_spectra_annotation/287-1-3-1-6-1.pdf]

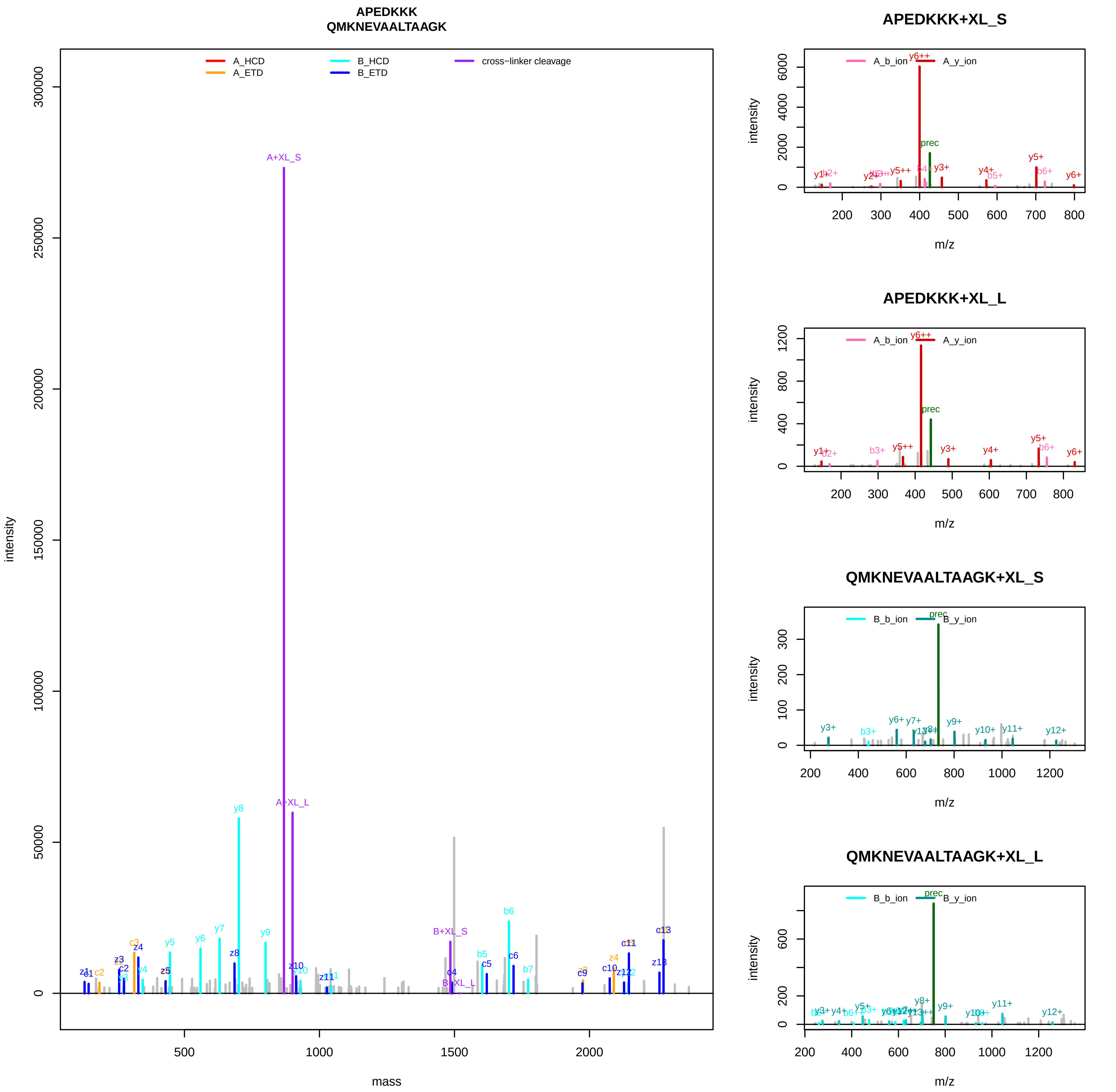
APEDKKK
QMKNEVAALTAAGK
A_HCD
A_ETD
B_HCD
B_ETD
cross−linker cleavage
A+XL_S
A+XL_L
B+XL_S
B+XL_L
y5
y6
y7
y8
y9
z4
z8
z10
z11
c3
c4
c5
c6
c9
c10
c11
c13
z13
b5
b6
b7
0
50000
100000
150000
200000
250000
300000
intensity
500
1000
1500
2000
mass
APEDKKK+XL_S
A_b_ion
A_y_ion
y6++
prec
y5++
y3+
y4+
b5+
y5+
b6+
y6+
y1+
y2+
0
2000
4000
6000
intensity
200
300
400
500
600
700
800
m/z
APEDKKK+XL_L
A_b_ion
A_y_ion
y6++
prec
b3+
y5++
y3+
y4+
y5+
b6+
y6+
y1+
b2+
0
400
800
1200
intensity
200
300
400
500
600
700
800
m/z
QMKNEVAALTAAGK+XL_S
B_b_ion
B_y_ion
prec
y3+
b3+
y6+
y7+
y9+
y10+
y11+
y12+
0
100
200
300
intensity
200
400
600
800
1000
1200
m/z
QMKNEVAALTAAGK+XL_L
B_b_ion
B_y_ion
prec
y3+
y4+
b6+
y5+
y8+
y9+
y11+
y12+
0
200
600
intensity
200
400
600
800
1000
1200
m/z
